# Multiparametric PET/CT-perfusion does not add significant additional information for initial staging in lung cancer compared with standard PET/CT

**DOI:** 10.1186/2191-219X-4-6

**Published:** 2014-01-22

**Authors:** Martin W Huellner, Timothy D Collen, Philipp Gut, Ralph Winterhalder, Chantal Pauli, Joachim Diebold, Burkhardt Seifert, Klaus Strobel, Patrick Veit-Haibach

**Affiliations:** 1 Department of Radiology and Nuclear Medicine, Lucerne Cantonal Hospital, Spitalstrasse 1, Lucerne CH-6004, Switzerland; 2 Department of Radiation Oncology, Lucerne Cantonal Hospital, Spitalstrasse 1, Lucerne CH-6004, Switzerland; 3 Clinic of Medical Oncology, Lucerne Cantonal Hospital, Spitalstrasse 1, Lucerne CH-6004, Switzerland; 4 Institute of Pathology, Lucerne Cantonal Hospital, Spitalstrasse 1, Lucerne CH-6004, Switzerland; 5 Division of Biostatistics, Institute for Social and Preventive Medicine, University of Zurich, Hirschengraben 84, Zurich CH-8001, Switzerland; 6 Department of Medical Radiology, Division of Nuclear Medicine, University Hospital Zurich, Rämistrasse 100, Zurich CH-8091, Switzerland; 7 Department of Medical Radiology, Institute of Neuroradiology, University Hospital Zurich, Rämistrasse 100, Zurich CH-8091, Switzerland; 8 Department of Medical Radiology, Institute of Diagnostic and Interventional Radiology, University Hospital Zurich, Rämistrasse 100, Zurich CH-8091, Switzerland; 9 Department of Pathology, University Hospital Zurich, Schmelzbergstrasse 12, Zurich CH-8091, Switzerland

**Keywords:** CT-perfusion, Lung cancer, FDG-PET/CT, Multimodality imaging, Functional imaging

## Abstract

**Background:**

The purpose of this study was to assess the relationship of CT-perfusion (CTP), ^18^F-FDG-PET/CT and histological parameters, and the possible added value of CTP to FDG-PET/CT in the initial staging of lung cancer.

**Methods:**

Fifty-four consecutive patients (median age 65 years, 15 females, 39 males) with suspected lung cancer were evaluated prospectively by CT-perfusion scan and ^18^F-FDG-PET/CT scan. Overall, 46 tumors were identified. CTP parameters blood flow (BF), blood volume (BV), and mean transit time (MTT) of the tumor tissue were calculated. Intratumoral microvessel density (MVD) was assessed quantitatively. Differences in CTP parameters concerning tumor type, location, PET positivity of lymph nodes, TNM status, and UICC stage were analyzed. Spearman correlation analyses between CTP and ^18^F-FDG-PET/CT parameters (SUV_max_, SUV_mean_, PET_vol_, and TLG), MVD, tumor size, and tumor stage were performed.

**Results:**

The mean BF (mL/100 mL min^-1^), BV (mL/100 mL), and MTT (s) was 35.5, 8.4, and 14.2, respectively. The BF and BV were lower in tumors with PET-positive lymph nodes (*p* = 0.02). However, the CTP values were not significantly different among the N stages. The CTP values were not different, depending on tumor size and location. No significant correlation was found between CTP parameters and MVD.

**Conclusions:**

Overall, the CTP information showed only little additional information for the initial staging compared with standard FDG-PET/CT. Low perfusion in lung tumors might possibly be associated with metabolically active regional lymph nodes. Apart from that, both CTP and ^18^F-FDG-PET/CT parameter sets may reflect different pathophysiological mechanisms in lung cancer.

## Background

Lung cancer is a major public healthcare burden for decades. Tobacco smoking accounts for 80% of lung cancers in males worldwide and for 50% of lung cancers in females [1-3]. In 2008, lung cancer was the most commonly diagnosed cancer and the leading cause of cancer-related death in men worldwide [3]. From a clinical point of view, early detection strategies have come into the focus, questioning if computed tomography (CT) screening of individuals with risk factors is beneficial [4-6]. While curative surgery is limited to the early stages of lung cancer, radiation therapy and chemotherapy are the treatment of choice in more advanced stages. Angiogenesis, as one of the key factors of tumor evolution and metastatic capability, can be assessed histopathologically by determining the microvessel density (MVD) [7]. Recently, new agents with antiangiogenic properties targeting tumor vascularity have been introduced [8-11].

Staging and restaging of lung cancer is nowadays usually done by ^18^F-fluoro-2-deoxy-d-glucose positron emission tomography/computed tomography (^18^F-FDG-PET/CT), which assesses cellular glucose metabolism. While the response to antiangiogenic agents in general may be assessed accurately by ^18^F-FDG-PET/CT, it is still not a direct measure of actual perfusion.

For non-invasive *in vivo* assessment of tumor vascularity, CT-perfusion (CTP) is a promising modality [12-14]. It is known that lung tumors with higher perfusion are more sensitive to radiochemotherapy than those with lower perfusion [13]. CTP has technical advantages over MR-perfusion in the lungs, concerning quantification of perfusion, motion artifacts, reproducibility, and resolution. Besides some recent studies [15,16], data about perfusion of the different types of lung cancer, being properly assessed by sufficient scan coverage and post-processing with motion-correction techniques, is still limited. Also, the relationship between CTP parameters, tumor size and stage, tumor location, ^18^F-FDG-PET/CT parameters, and MVD is partly unclear. Thus, the aims of our study were (1) to assess the CTP and ^18^F-FDG-PET/CT parameters in different subtypes of lung cancer and their possible contribution to staging and (2) to analyze the relationship between the morphological, functional, and metabolic parameters in these different subtypes.

## Methods

### Patients

This prospective study was approved by the Institutional Review Board and by the Cantonal Ethics Committee. All patients provided informed signed consent prior to the examinations.

Between November 2010 and December 2011, 54 consecutive patients (median age 65 years, range 42 to 79 years, 15 females, 39 males) with suspected lung cancer being referred for baseline staging PET/CT were evaluated prospectively. All patients received a CT-perfusion scan of the lung tumor after a partial-body ^18^F-FDG-PET/CT scan within 15 min at our institution. The exclusion criteria were as follows: renal insufficiency (renal clearance below 30 mL/min) without dialysis, known allergy or hypersensitivity to iodinated contrast medium, untreated hyperthyroidism, pregnancy, and non-compliance with recommended 6-h fasting period before PET/CT. Eight patients were excluded after the scan because histology results revealed lesions other than lung cancer. Finally, 46 patients were eligible for analysis. If the pathological TNM (pTNM) was not available as standard of reference in a patient, e.g., because that patient underwent primary radiochemotherapy, clinical TNM (cTNM) was employed. The cTNM was derived by imaging, transthoracic and transbronchial biopsy/mediastinoscopy. Histopathological determination of the N status was always forced in case there was no clear metastatic involvement of regional lymph nodes on imaging, such as nodes with necrotic centers and FDG-avid rims.

### CT imaging protocols

All CTP scans were performed on a 256-slice CT scanner (Somatom Definition Flash, Siemens Healthcare, Erlangen, Germany). The covered *z*-axis scan length was 7 cm (4D range). This relatively large scan length was chosen to cover the whole tumor in every patient. The fixed tube current was 100 mAs, and the fixed tube voltage was 100 kV (peak). The duration of the CTP scan was 60 s, with a rotation time of 1 s. CT-perfusion scanning was delayed by 3 s after the start of the injection of 40 mL of contrast medium (CM; Ultravist 370, Bayer Healthcare, Leverkusen, Germany) at 4.5 mL/s. The CM was injected into an antecubital vein by a dual-head pump injection device (Stellant D, Medrad, Warrendale, PA, USA), followed by a flush of 50 mL of NaCl at 4.5 mL/s. With such a rather lengthy protocol, high reproducibility of perfusion parameters can be obtained [17].

The patients were advised to resume shallow breathing for the entire duration of the scan. The collimation was 64 × 0.6 mm. The CT-perfusion reconstruction increment was 3 mm at 5-mm slice width. Image reconstruction was performed with a 512 × 512-pixel matrix and medium smooth B30f kernel. For image post-processing and analysis, the reconstructed images were transferred to a commercially available computer workstation (*syngo* Multimodality Workplace, Siemens Healthcare).

### ^18^F-FDG-PET/CT imaging protocols

All PET/CT scans were performed on a combined in-line system (Discovery PET/CT 600, GE Healthcare, Milwaukee, WI, USA) with a multidetector helical 16-slice CT and integrated full-ring PET. This dedicated system allows for acquisition of co-registered PET and CT images in one step. After the injection of a standard dose of 300 to 340 MBq ^18^F-FDG, the PET/CT imaging started with a delay of 60 min. The patients were advised to drink 1,000 ml of oral contrast medium during this uptake time.

The non-enhanced low-dose CT part of the combined scan was acquired with a tube voltage of 120 kV, a tube current of 40 mA, and a tube rotation time of 0.5 s. The imaging range was from the vertex to the upper thighs. Consecutively, the emission PET data acquisition started with an acquisition time of 2 min per bed position. The CT data was used for attenuation correction. CT images were later reconstructed with 3.75-mm slice width, using a fully 3D iterative algorithm (ordered subset expectation maximization (OSEM)). For image post-processing, co-registration, and analysis, the reconstructed images were transferred to a commercially available computer workstation (Advantage Workstation 4.4, GE Healthcare).

### Image evaluation

All evaluations were performed as a lesion-based analysis by two experienced radiologists in consensus. CT perfusion parameters blood flow (BF), blood volume (BV), and mean transit time (MTT) were determined by post-processing on the workstation, using a dedicated lung tumor preset of a perfusion evaluation software (*syngo*^®^ Volume Perfusion CT Body, Siemens Healthcare). A dataset motion correction and a noise reduction algorithm were applied automatically. The processing thresholds or segmentation tissue limits were -50 and 150 HU to exclude bone and other hyperdense materials. The window width and center for the reference vessel input was 300 and 150 HU, respectively. The relative threshold for inside and outside was 50%; an adaptive smoothing filter was used. The vendor’s default standard algorithmic parameters were applied. 3D color-coded maps for BF, BV, and MTT were generated with a sequential two-compartment model (modified Patlak approach). Blood flow (mL/100 mL min^-1^) is defined as the amount of blood flowing through 100 mL of tumor tissue within 1 min. Mean transit time (s) is defined as the average time of contrast agent residence within the tumor tissue. Blood volume (mL/100 mL) is the product of BF and MTT and is defined as the amount of blood within 100 mL of tumor tissue. BV can be expressed as the proportion of the total volume of a dedicated voxel. For every patient, an individual arterial input fraction was determined by placing an analytic region of interest (ROI) into the pulmonary trunk, if depicted, or into the ipsilateral pulmonary artery. A dedicated free-hand volume of interest (VOI) was drawn around the whole tumor in the lung window and adapted to its borders, trying to exclude necrotic tumor areas, in all three planes to ensure reliable perfusion measurements [18]. Adjacent bones, bronchi, and soft tissue structures were excluded. The mean values of the CTP parameters were recorded for the whole-tumor VOI.

The total tumor size (mm) was measured on the CT images in the lung window preset on a commercially available picture archiving and communication system (PACS) workstation (Merlin Diagnostic Workcenter 4.1, Phönix-PACS, Freiburg, Germany). The tumor location was determined vertically (upper vs. lower lung) and radially (central vs. peripheral lung).

The PET/CT images were evaluated on a workstation which permits multiplanar reconstruction as single (CT only, PET only) and combined (co-registered PET/CT) procedures. For study purposes, the maximum (SUV_max_) and the mean standardized uptake values (SUV_mean_) of the tumor, the metabolic tumor volume (PET_vol_), and the total lesion glycolysis (TLG; PET_vol_ × SUV_mean_) were determined. All the SUV assessments were corrected for body weight and height. All the PET/CT images were evaluated by a dual-board-certified nuclear physician/radiologist in clinical routine for staging according to our institution’s clinical protocols.

### Histopathological analysis

To characterize the tumor vasculature, the mean intratumoral MVD was quantitatively assessed according to immunohistochemical CD34 staining. We obtained formalin-fixed specimens embedded in paraffin of the 15 patients who underwent surgical tumor resection by thoracotomy. The specimens were cut into 4-μm slices and fixed to histology slides (X-tra, Leica Biosystems, Nussloch, Germany). After hematoxylin/eosin staining, the slides were stained with CD34 antibody (1:30, NCL-L END, Novocastra, Leica Biosystems, Nussloch, Germany) using an automated staining system (BenchMark XT, Ventana Medical Systems, Oro Valley, AZ, USA). The slides were scanned with a slide scanner (iScan Coreo, Ventana Medical Systems). A pathologist blinded to clinical and imaging data performed the histopathological analysis by visually counting the positive microvessels on the scanned images using public domain software (imageJ, http://rsbweb.nih.gov/ij).

### Statistical analysis

Comparisons of the CTP parameters, ^18^F-FDG-PET/CT parameters, tumor size, and MVD values were evaluated by Mann–Whitney *U* test or Kruskal-Wallis test for subtypes of tumors as defined by histology. The results were illustrated as box plots. The correlations between pairs of parameters (CTP, ^18^F-FDG-PET/CT, size, stage, and MVD) were evaluated by Spearman’s rank correlation coefficient due to the skew distribution of the data. The results were interpreted as strong correlation between ±0.5 and ±1.0, moderate between ±0.3 and ±0.49, weak between ±0.1 and ±0.29, and no correlation below ±0.1 [19]. The bootstrap method was used for linear regression analysis of the relationship between the dependent variables (SUV_max_, SUV_mean_, PET_vol_, TLG, MVD, and size) and the independent variables (BF, BV, and MTT). A *p* value of <0.05 was considered statistically significant. The software employed was IBM SPSS Statistics™ 19.0.1 (SPSS Inc., Chicago, IL, USA).

## Results

**Figure 1:**
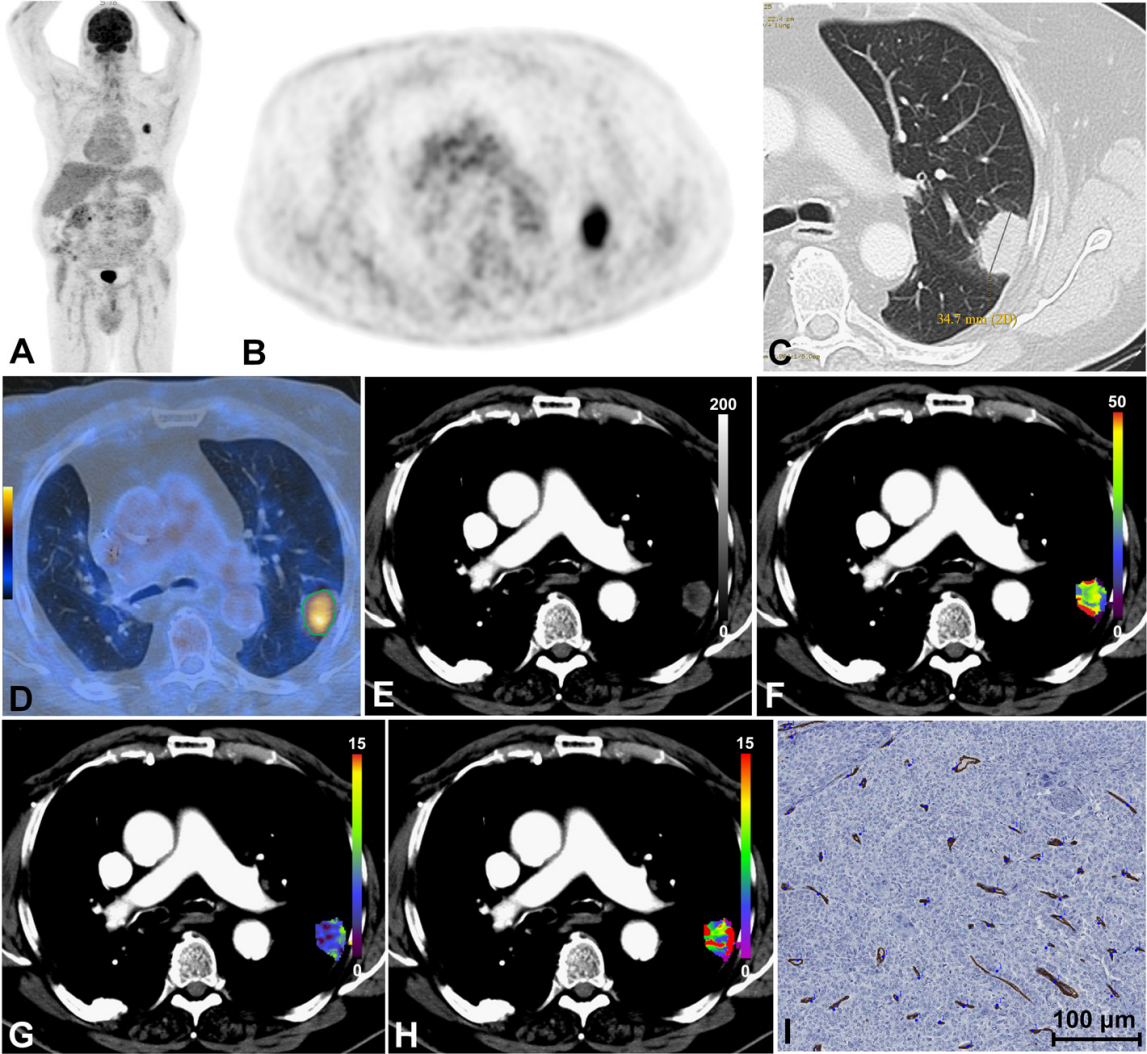
**Patient with peripheral adenocarcinoma of the left lower lobe. (A)** Coronal PET-MIP, **(B)** axial PET, **(C)** axial CT in lung window, and **(D)** axial ^18^F-FDG-PET/CT co-registration. The green VOI encompasses the metabolic tumor volume with 42% isocontour. The tumor is 34.7 mm in size and exhibits a SUV_max_ of 7.1, SUV_mean_ of 4.6, TLG of 54.9, and PET_vol_ of 11.8 cm^3^. **(E)** Axial time MIP. Color-coded maps for **(F)** BF, **(G)** BV, and **(H)** MTT. The tumor has a BF of 29.8 mL/100 mL min^-1^, a BV of 7.1 mL/100 mL, and an MTT of 12.2 s. **(I)** MVD analysis of the post-surgical specimen stained in hematoxylin/eosin, detected vessels labelled blue. ^18^F-FDG-PET/CT, ^18^F-fluoro-2-deoxy-d-glucose positron emission tomography/computed tomography; BF, blood flow; BV, blood volume; CT, computed tomography; MVD, microvessel density; MTT, mean transit time; MIP, maximum intensity projection; PET, positron emission tomography; PET_vol_, metabolic tumor volume; SUV_max_, maximum standardized uptake value; SUV_mean_, mean standardized uptake value; TLG, total lesion glycolysis; VOI, volume of interest.

The majority of the patients were diagnosed with non-small cell lung carcinoma (NSCLC; *n* = 41), and five patients with small cell lung carcinoma (SCLC). The NSCLC group consisted of adenocarcinoma (AC; *n* = 24, Figure 1A,B,C,D,E,F,G,H), squamous cell carcinoma (SCC; *n* = 9), large cell carcinoma (LCC; *n* = 7), and one neuroendocrine tumor (NET). The results derived from CTP, ^18^F-FDG-PET/CT, tumor size and stage assessment, and histopathological analysis were stratified according to these histologic subtypes. During their consecutive therapy, 15 patients (all with NSCLC, thereof nine adenocarcinomas, five squamous cell carcinomas, one large cell carcinoma) underwent curative surgery and adjuvant radiochemotherapy, providing pTNM status and MVD for analysis. Fifteen patients underwent primary curative radiochemotherapy, 13 palliative chemotherapy, and 3 neoadjuvant therapy and surgery. In these 31 patients, only cTNM was available.

### Descriptive statistics

**Figure 2:**
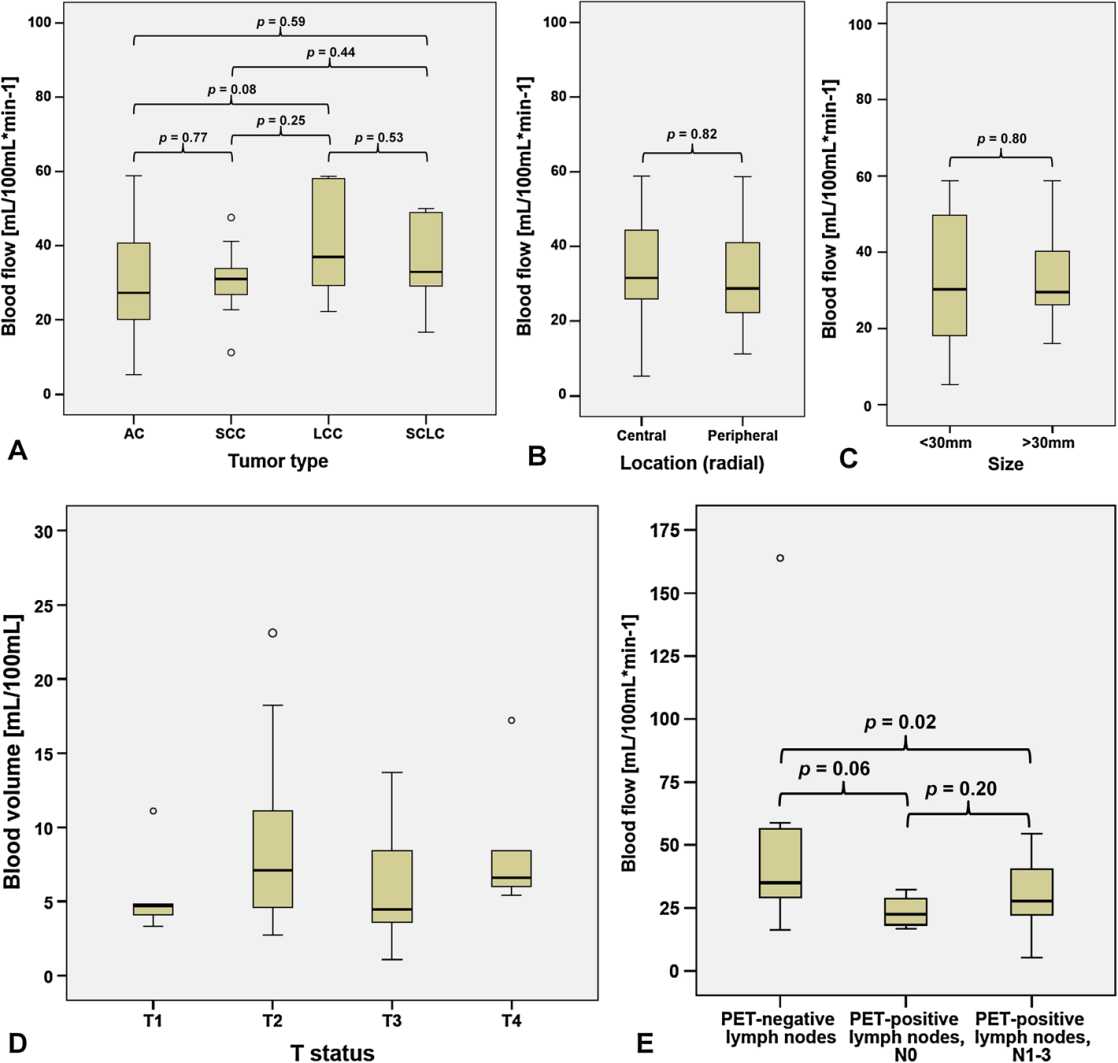
**Boxplots of CTP parameters, clinical tumor parameters, and **^**18**^**F-FDG-PET/CT parameters. (A)** BF in different tumor types. **(B)** BF according to radial location of tumor. **(C)** BF according to tumor size. **(D)** BV according to T status. **(E)** BF according to PET positivity of regional lymph nodes stratified by N status. PET-negative *n* = 17, PET-positive/N0 *n* = 4, PET-positive/N1 to N3 *n* = 25. Circle depicts outlier. BF, blood flow; BV, blood volume; CT, computed tomography; CTP, CT-perfusion; ^18^ F-FDG-PET/CT, ^18^F-fluoro-2-deoxy-d-glucose positron emission tomography/computed tomography; PET, positron emission tomography.

The anatomical and clinical characteristics, CT-perfusion and ^18^F-FDG-PET/CT data, and MVD are summarized in Table 1 for all the tumors. The overall average tumor size was 41.1 mm. No significant differences were found for the TNM status and UICC stage. CT-perfusion parameters did not differ significantly between the tumor types (Figure 2A). The mean BF was 35.5 mL/100 mL min^-1^ in NSCLC and SCLC, the mean BV was 8.4 mL/100 mL and 8.6 mL/100 mL, respectively, and the mean MTT 14.4 and 12.4 s, respectively. The average SUV_max_ of all the tumors was 11.7, SUV_mean_ 7.2, TLG 120, and PET_vol_ 17.0 cm^3^. Also, the ^18^F-FDG-PET/CT parameters did not differ significantly between NSCLC and SCLC. Among the NSCLC subtypes, significant differences were found concerning the location and T status. While AC and SCC were located preferably in the upper parts of the lung, LCC yielded a balanced vertical distribution (*p* = 0.008). Lower T stages dominated in AC and LCC, whereas the median T stage in SCC was T3 (*p* = 0.008). CT-perfusion and ^18^F-FDG-PET/CT parameters and MVD did not differ significantly among the NSCLC subtypes.

**Table 1:** **Anatomical and clinical CT-perfusion and **^**18**^**F-FDG-PET/CT characteristics of the histologic lung cancer subtypes**

**Tumor parameters**	**All tumors (*n*** **= 46)**	**Tumor type**			**NSCLC subtype**^**b**^			
		**SCLC (*n*** **= 5)**	**NSCLC (*n*** **= 41)**	***p***	**Adenocarcinoma (*n*** **= 24)**	**Squamous cell carcinoma (*n*** **= 9)**	**Large cell carcinoma (*n*** **= 7)**	***p***
Anatomical characteristics								
Longest diameter (mm), mean ± SD	41.1 ± 20.1	35.2 ± 18.5	42.0 ± 20.9	0.70	39.9 ± 20.9	45.8 ± 21.3	37.0 ± 9.9	0.80
Location superior/inferior	83%/17%	80%/20%	83%/17%	0.92	87%/13%	100%/0%	43%/57%	0.008
Location central/peripheral	43%/57%	80%/20%	39%/61%	0.14	33%/66%	66%/33%	29%/73%	0.18
Clinical characteristics								
T stage (median (range))	T2a (T1 to T4b)	T2a (T1b to T4)	T2a (T1a to T4)	0.84	T2a (T1a to T4)	T3 (T2a to T4)	T2a (T1b to T2b)	0.008
T1	5	1	4		2	0	2	
T2	29	2	27		18	4	5	
T3	6	1	5		3	2	0	
T4	6	1	5		1	3	0	
N stage (median (range))	N1 (N0 to N3)	N2 (N0 to N3)	N1 (N0 to N3)	0.47	N2 (N0 to N3)	N1 (N0 to N3)	N0 (N0 to N3)	0.41
N0	15	1	13		7	1	4	
N1	10	0	11		4	6	1	
N2	9	3	6		5	0	1	
N3	12	1	11		8	2	1	
M stage (median (range))	M0 (M0 to M1)	M0 (M0)	M0 (M0 to M1)		M0 (M0 to M1)	M0 (M0 to M1)	M1 (M0 to M1)	0.89
M0	28	5	23	0.41	14	6	3	
M1	18	0	18		10	3	4	
UICC stage (median (range))	IIIB (IA to IV)	IIIA (IIB to IIIB)	IIIB (IA to IV)	0.32	IIIB (IA to IV)	IIIA (IIA to IV)	IV (IB to IV)	0.88
CT-perfusion characteristics								
Blood flow (mL/100 mL min^-1^), mean ± SD	35.5 ± 23.5	35.5 ± 12.6	35.5 ± 24.3	0.67	31.4 ± 14.1	30.5 ± 9.8	56.9 ± 45.7	0.21
Blood volume (mL/100 mL), mean ± SD	8.4 ± 6.4	8.6 ± 4.8	8.4 ± 6.4	0.85	9.1 ± 7.4	5.3 ± 2.8	9.9 ± 5.4	0.16
Mean transit time (s), mean ± SD	14.2 ± 4.5	12.4 ± 3.8	14.4 ± 4.5	0.45	14.6 ± 4.4	16.3 ± 4.1	11.8 ± 4.0	0.30
^18^F-FDG-PET/CT characteristics								
SUV_max_ (mean ± SD)	11.7 ± 5.5	9.1 ± 3.0	12.0 ± 5.6	0.35	11.3 ± 4.9	14.3 ± 5.8	9.7 ± 4.8	0.28
SUV_mean (42%)_ (mean ± SD)	7.2 ± 3.3	5.8 ± 1.7	7.4 ± 3.5	0.43	6.9 ± 2.9	8.9 ± 3.8	6.1 ± 3.0	0.29
TLG (median (range))	120.0 (1.9 to 2621)	87.8 (7.8 to 232)	124.3 (1.9 to 2621)	0.39	116.2 (1.9 to 1,900)	124.3 (30.5 to 1,555)	101.5 (33.2 to 243.5)	0.88
PET_vol_ (cm^3^) (median (range))	17.0 (1.2 to 315)	12.6 (3.1 to 39.6)	18.2 (1.2 to 315)	0.58	18.1 (1.2 to 315)	14.1 (3.4 to 68.0)	18.2 (4.3 to 67.7)	0.93
PET-positive lymph nodes	63%	100%	59%	0.14	64%	67%	43%	0.59
Microvessel density^a^ (1/mm^2^), mean ± SD	149.9 ± 79.7	-	149.9 ± 79.7	-	155.8 ± 75.9	153.7 ± 96.4	77.1	0.50

^18^F-FDG-PET/CT, ^18^F-fluoro-2-deoxy-d-glucose positron emission tomography/computed tomography; CT, computed tomography; NSCLC, non-small cell lung carcinoma; PET, positron emission tomography; PET_vol_, metabolic tumor volume; SD, standard deviation; SCLC, small-cell lung carcinoma, SUV_max_, maximum standardized uptake value; SUV_mean_, mean standardized uptake value; TLG, total lesion glycolysis; UICC, Union for International Cancer Control. ^a^Microvessel density data only available from 15 patients (15 NSCLC, thereof 9 adenocarcinoma, 5 squamous cell carcinoma, 1 large cell carcinoma). ^b^Without the single neuroendocrine tumor.

### CT-perfusion parameters by location, TNM status, UICC stage, and PET positivity of lymph nodes

For further characterization of the CT-perfusion results, subanalyses concerning the size, location, TNM status (seventh edition), and UICC stage (seventh edition) of the tumors, and the PET positivity of the regional lymph nodes were done (Tables 2 and 3). The CT-perfusion parameters were not significantly different, depending on the vertical (upper vs. lower lung) or radial location (central vs. peripheral lung) (Figure 2B), or size (Figure 2C). No significant difference was found concerning the T status and M status (Figure 2D). The BV was significantly different, depending on the N status, with higher values obtained in N0 and N2 tumors, and lower values in N1 and N3 tumors (*p* = 0.005). The UICC stages yielded no significant difference between the CT-perfusion parameters. The tumors in patients displaying PET-positive regional lymph node metastases had significantly lower BF and BV (all tumors: *p* = 0.02; NSCLC: *p* = 0.01) than the tumors in patients with PET-negative nodes (Figure 2E).

**Table 2:** CT-perfusion parameters by TNM status (seventh edition)

**CT-perfusion parameters**	**T status**					**N status**					**M status**		
**All tumors ( *n * = 46)**	**T1 ( *n * = 5)**	**T2 ( *n * = 29)**	**T3 (*n*** **= 6)**	**T4 (*n*** **= 6)**	***p***	**N0 ( *n * = 15)**	**N1 ( *n * = 10)**	**N2 (*n*** **= 9)**	**N3 (*n*** **= 12)**	***p***	**M0 ( *n * = 28)**	**M1 (*n*** **= 18)**	***p***
Blood flow (mL/100 mL min^-1^), mean ± SD	32.1 ± 12.7	38.2 ± 28.3	28.3 ± 10.9	32.3 ± 9.5	0.81	45.6 ± 36.2	27.1 ± 10.9	35.7 ± 9.2	29.7 ± 13.9	0.14	37.6 ± 28.4	32.2 ± 12.9	0.92
Blood volume (mL/100 mL), mean ± SD	5.6 ± 3.1	9.4 ± 7.3	6.0 ± 4.5	8.4 ± 4.4	0.26	11.3 ± 8.9	4.7 ± 2.8	10.5 ± 4.9	6.3 ± 2.7	0.005	8.4 ± 7.0	8.4 ± 5.4	0.73
Mean transit time (s), mean ± SD	14.4 ± 2.6	13.6 ± 4.8	14.8 ± 4.2	15.9 ± 5.1	0.78	13.5 ± 5.9	14.7 ± 2.9	15.0 ± 4.2	13.9 ± 4.2	0.48	13.5 ± 4.7	15.2 ± 4.1	0.39
**NSCLC (*n =*** **41)**	**T1 ( *n * = 4)**	**T2 ( *n * = 27)**	**T3 ( *n * = 5)**	**T4 ( *n * = 5)**	***p***	**N0 ( *n * = 14)**	**N1 ( *n * = 10)**	**N2 ( *n * = 6)**	**N3 ( *n * = 11)**	***p***	**M0 ( *n * = 26)**	**M1 ( *n * = 15)**	***p***
Blood flow (mL/100 mL min^-1^), mean ± SD	32.0 ± 14.7	38.2 ± 29.3	30.6 ± 10.4	28.3 ± 10.9	0.96	47.7 ± 36.7	27.1 ± 10.9	34.9 ± 9.2	27.9 ± 13.1	0.08	37.4 ± 29.4	32.2 ± 12.7	0.89
Blood volume (mL/100 mL), mean ± SD	6.0 ± 3.5	9.5 ± 7.5	6.2 ± 4.9	6.0 ± 4.5	0.57	11.9 ± 9.0	4.7 ± 2.8	10.4 ± 4.6	6.2 ± 2.8	0.003	8.6 ± 7.2	8.0 ± 5.3	0.95
Mean transit time (s), mean ± SD	13.8 ± 2.6	13.8 ± 4.7	15.5 ± 4.3	16.6 ± 5.4	0.65	13.6 ± 6.1	14.7 ± 2.9	15.1 ± 5.1	14.7 ± 3.6	0.62	13.7 ± 4.6	15.6 ± 4.3	0.30

CT, computed tomography; NSCLC, non-small cell lung carcinoma; SD, standard deviation.

**Table 3:** CT-perfusion parameters by UICC stage (seventh edition) and PET positivity of lymph nodes

**CT-perfusion parameters**	**UICC stage**					**PET-positive lymph nodes**		
**All tumors ( *n * = 46)**	**I ( *n * = 6)**	**II ( *n * = 10)**	**III (*n*** **= 12)**	**IV ( *n * = 18)**	***p***	**No (*n*** **= 17)**	**Yes ( *n * = 29)**	***p***
Blood flow (mL/100 mL min^-1^), mean ± SD	30.0 ± 17.8	42.0 ± 44.4	37.8 ± 13.0	32.2 ± 12.9	0.61	45.7 ± 33.6	29.5 ± 12.0	0.02
Blood volume (mL/100 mL), mean ± SD	7.3 ± 6.1	7.5 ± 4.4	9.7 ± 9.2	8.4 ± 5.4	0.82	11.1 ± 8.4	6.8 ± 4.1	0.02
Mean transit time (s), mean ± SD	12.8 ± 5.8	13.8 ± 4.2	13.7 ± 5.0	15.2 ± 4.1	0.79	12.9 ± 4.6	14.9 ± 4.4	0.09
**NSCLC ( *n * = 41)**	**I ( *n * = 6)**	**II ( *n * = 9)**	**III ( *n * = 11)**	**IV ( *n * = 15)**	***p***	**No ( *n * = 17)**	**Yes ( *n * = 24)**	***p***
Blood flow (mL/100 mL min^-1^), mean ± SD	30.0 ± 17.8	43.0 ± 47.0	37.8 ± 13.0	32.2 ± 12.9	0.70	45.7 ± 33.6	28.3 ± 11.5	0.01
Blood volume (mL/100 mL), mean ± SD	7.3 ± 6.1	7.8 ± 4.5	10.0 ± 9.6	8.0 ± 5.3	0.84	11.1 ± 8.4	6.5 ± 3.9	0.01
Mean transit time (s), mean ± SD	12.8 ± 5.8	13.5 ± 4.3	14.3 ± 4.6	15.6 ± 4.3	0.72	12.9 ± 4.6	15.4 ± 4.3	0.04

CT, computed tomography; NSCLC, non-small cell lung carcinoma; PET, positron emission tomography; SD, standard deviation; UICC, Union for International Cancer Control.

### Correlation analysis

Based on the results of Spearman’s rank correlation coefficient (*r*) analysis of the CT-perfusion parameters, ^18^F-FDG-PET/CT parameters, MVD, and tumor size and stage, the following correlations were found: In the LCC group, strong inverse correlations between BF and SUV_max_ and SUV_mean_ are represented by a Spearman’s correlation coefficient of -0.86 (*p* = 0.01) each. BV yielded a strong positive correlation with the T status of LCC (*r* = 0.79, *p* = 0.03). MTT rendered a strong correlation with the N status and the UICC stage in LCC (*r* = 0.91, *p* = 0.005; *r* = 0.81, *p* = 0.03; respectively) and a moderate correlation with UICC stage in SCC (*r* = 0.32, *p* = 0.05). In the numerically largest NSCLC subgroup AC, no correlation was found between the CT-perfusion parameters and the other parameters. Besides, CT-perfusion parameters did not yield a significant correlation with MVD in any group.

## Discussion

We evaluated the relationship of CTP and the ^18^F-FDG-PET/CT parameters, and the possible additional value of CTP in an initial staging setting in lung cancer. Up to date, there is only little knowledge about these relationships available in the literature. Our main result is that CTP does not add significant information concerning the staging of lung cancer. Only one questionable correlation between FDG-positive lymph node metastases and perfusion values, as well as several indicative correlations for the small group of LCC, was found. Overall, CTP does possibly reflect different pathophysiological pathways than ^18^F-FDG-PET/CT.

The introduction of combined functional imaging such as PET/CT has changed the staging and therapy response evaluation in lung cancer patients. While morphological tumor assessment by CT provides information about structure and extent, ^18^F-FDG-PET/CT facilitates the perception of the metabolic activity. CT-perfusion imaging, however, provides information about the vasculature of tissue as expressed by, e. g., blood flow and blood volume. The value of the combined assessment of tumor flow and metabolism in terms of a flow-metabolic characterization was shown to provide additional diagnostic information for tumor grading and prediction of treatment response in breast cancer, pancreatic cancer, and colorectal cancer [20-26]. However, in our present study on lung cancer, overall, only little additional significant information concerning initial staging could be demonstrated.

### Tumor type and perfusion-metabolism relationship

Knowledge of the relationship of tumor perfusion and metabolism could provide new insights into its biological and pathophysiological characteristics, perhaps more than either method alone [27]. Data on the perfusion-metabolism relationship in lung cancer is limited and somewhat erratic. Most of the CTP literature on lung cancer does not differentiate between the NSCLC subgroups, probably due to small study cohorts, and only a few studies focus on the perfusion-metabolism relationship. A recent study by Sauter et al. discriminates between AC and SCC [16]. They showed that the CTP parameters are not different in these two groups, which parallels our results and of the previous [21]. However, they did not provide a correlation analysis between the CTP and ^18^F-FDG-PET/CT parameters. Schmid-Bindert et al. found a strong direct correlation between SUV_max_ and maximum iodine attenuation in dual-energy CT (DECT) in NSCLC [28], which is however not directly comparable with perfusion parameters.

Ippolito et al. discriminate a larger number of NSCLC (*n* = 29) and a very small number of SCLC (*n* = 3) and state that there is no significant difference in the CTP parameters among those [29]. However, these numbers are small, and a subanalysis for the NSCLC group is not provided. In the present study, we obtained CTP values similar to those obtained by Sauter et al., but in part very different from those by Ippolito et al. (e. g., NSCLC: mean BF 35.5, 35.8, and 111.6 mL/100 mL min^-1^, mean BV 8.4, 8.1, and 6.0 mL/100 mL (present study, Sauter et al., Ippolito et al., respectively)) [16,29]. One reason may be refraining from motion correction by Ippolito et al. [29]. Motion leads to falsely high BF values at the lung/tumor interface (see also ‘Reproducibility’ section below). However, they performed a regression analysis for the perfusion and metabolism parameters and stated a weak but significant inverse linear relationship (*r*^*2*^ = 0.21) between the BF and SUV_max_ and a direct linear relationship (*r*^*2*^ = 0.23) between the MTT and SUV_max_ in tumors larger than 3 cm [29]. We found such an inverse correlation between the BF and metabolism parameters SUV_max_ and SUV_mean_ only for the LCC subgroup (*r* = -0.86), which might however be due to the rather small number of LCC in our cohort. LCC are usually tumors of poor differentiation and high malignant potential. They exhibit aggressive biological features and poor survival rates. Such tumors typically have partly hypoxic areas, and hypoxia may be associated with FDG uptake and aggressiveness in some malignant tumors [30]. Matched high glucose metabolism with increased vascularity (coupling) represents a different biological status as compared with mismatched high metabolism and low vascularity, the latter possibly indicating adaptation to hypoxia [31,32]. If the recruitment of pathologic vessels is insufficient to sustain the tumor’s energy needs, then finally necrosis may result, and necrotic lung cancer is known to exhibit decreased perfusion [21]. For NSCLC subgroups other than LCC, such associations could not be shown. Since in our study the patient number is higher than in the other studies available in the literature and we could not demonstrate resilient general trends in the NSCLC group, such relationships might be debatable.

It is known that motion has a considerable effect not only on the CTP parameters, but also on the SUV. The observed lack of correlations between the perfusion and metabolism parameters in the majority of lesions could partly be due to the absence of motion correction of the PET images in our study.

In our entire study cohort, the tumors with PET-positive lymph node metastases showed significantly lower BF and BV than those without. This might be explained by necrotic areas in more aggressive or advanced tumors that have already spread to the regional lymph nodes. Such a possible correlation is partly supported by a recent study in which an inverse correlation between BF and SUV_mean_ (*r* = -0.51) and BF and SUV_max_ (*r* = -0.54) was found in the mediastinal lymph nodes [15]. Yet, these correlations were only observed for lymph nodes with a SUV_max_ > 2.5, PET/CT and CT perfusion were not done on the same day, and histopathological verification was incomplete. What challenges such possible correlations in our study is that the BF and BV of the few tumors with ‘false’ PET-negative nodes were not significantly different from tumors with ‘true’ PET-negative nodes. On the other hand, one large PET/CT study has shown that the FDG uptake by the primary tumor is an independent predictor of regional lymph node metastasis in patients with NSCLC [33]. Based on the histopathological results of our cohort, we can however not corroborate the presence of necrosis in tumors with regional lymph node metastases since such quantitative data was not acquired.

### Tumor stage, size, and location

The clinical tumor stage (UICC), T, and N status did not correlate with blood flow in the entire cohort (*n* = 46) or in NSCLC (*n* = 41). There were again only considerable correlations between MTT and tumor stage in certain subgroups such as SCC (*n* = 9, UICC stage) and LCC (*n* = 7, N stage). However, those subgroups were the smallest ones, and the actual MTT values were not significantly different if stratified by UICC stage and N stage.

The tumor size is one of the main determinants of the tumor stage. In an early study employing the maximum slope method in a mixed population of advanced NSCLC and SCLC, the perfusion was higher in smaller tumors [34]. For NSCLC in general, Miles et al. did not observe a significant correlation between SUV and standardized perfusion value (SPV; SPV = tissue perfusion/whole body perfusion); however, smaller tumors (<4.5 cm^2^) exhibited a strong correlation [27]. Yet, in a mixed group of nine small (<3 cm^2^) dedifferentiated lung tumors, an inverse correlation between enhancement, being assessed with dynamic contrast-enhanced (DCE) CT, and lesion diameter was observed [35]. Higher BV in tumors smaller than 3 cm was also found in a heterogeneous sample of peripheral lung cancer [36]. In a mixed cohort of mainly NSCLC, tumors larger than 3 cm exhibited a borderline significant tendency towards lower BF and BV, while SUV_max_ was not different [29]. However, the numbers were distributed unevenly across the analyzed groups. The authors concluded that larger and thus more aggressive tumors might have lower perfusion [29]. In our opinion, this needs further investigation since aggressive biological behavior is not merely a reflection of size. In our (larger) study, we found no significant differences in the CTP parameters between smaller (<30 mm) and larger tumors (>30 mm). Tumor size assessed in terms of diameter, according to RECIST 1.1, was also not correlated with the CTP or ^18^F-FDG-PET/CT parameters, neither in all tumors nor in any subgroup.

Gravity and patient position have an impact on lung perfusion. Pulmonary perfusion is different when assessed in the supine position as with CT, as opposed to the upright position, where there is three times more blood flow in the lung bases than in the apices. Studies found differences in perfusion with higher values obtained in lower-lobe tumors or higher perfusion in peripherally located tumors [29,34], which was not the case in our study. In the present study, no perfusion differences related to tumor size and location could be substantiated, and no significant information for staging could be demonstrated.

### Microvessel density

Microvessel density in NSCLC is associated with distant metastatic spread and poor survival [37]. In other tumors, there is evidence that the degree of vascularization decreases with the grade of differentiation [38]. Several previous studies have shown that DCE measurements of lung cancer correlate with the histopathological assessment of tumor vascularization such as MVD [36,39,40]. The failure to demonstrate a relationship between CTP parameters and MVD in the present study is likely due to our study setting, as we covered tumors of any size rather than focusing on small malignant nodules, which are usually removed surgically and qualify for MVD analysis, whereas larger tumors are often irradiated. Additionally, the association between CTP and MVD might also depend on the histopathological method used [41-44].

### Reproducibility

The reproducibility, and thus reliability, of quantitative whole lung tumor CTP was ascertained by Ng and et al. in ten patients with advanced NSCLC [45]. It was furthermore shown to improve with greater *z*-axis scan coverage [46] and motion correction [17]. Both sufficient scan coverage (7 cm) and state-of-the-art motion correction were part of our study. In another study, the CTP assessment was performed in breath-hold technique for 25 to 30 s [29]. In our opinion, the implementation of motion correction is mandatory. First, in our experience, there is a slight motion conceivable within the lung parenchyma even in so-called breath-hold, increasingly if prolonged, which inevitably influences perfusion values. Second, tumors located close to the heart are subject to its movement anyhow. Third, most patients will instinctively hold their breath in inspiration and thus perform a Valsalva maneuver. This has an impact on lung perfusion by increasing the intrathoracic pressure. Last, lung cancer patients are sometimes limited in their capability of breath-hold due to coexisting emphysema and also due to compromise by the tumor itself. This allows only short periods of breath-hold and may even result in additional incorrect measurement due to possible gasping prior to the end of the measurement.

### Limitations

The MVD analysis was only possible in one third of patients as the remaining patients did not qualify for surgery. Quantitative histopathological data about the presence of necrosis within tumors was not acquired. Most of the TNM stages reported were clinical as opposed to pathologic. The number of LC and SCC was quite small, and the results regarding these NSCLC subgroups should be interpreted with caution. We also did not acquire motion-corrected PET, which could have contributed to the lack of correlations in the majority of tumors.

## Conclusions

In the present study, CTP generated only little additional information for the initial staging of lung cancer compared with standard PET/CT. In lung cancer, there might be lower tumor perfusion in patients with metastatic lymph node spread. However, this relationship certainly needs further investigation. The possible association of CTP parameters with clinical tumor stage also needs confirmation from larger studies.

## Abbreviations

18F-FDG-PET/CT: ^18^F-fluoro-2-deoxy-d-glucose positron emission tomography/computed tomography; 3D: three-dimensional; 4D: four-dimensional; AC: adenocarcinoma; BF: blood flow; BV: blood volume; CD34: cluster of differentiation molecule 34; CM: contrast medium; CT: computed tomography; CTP: CT-perfusion; DECT: dual-energy CT; DCE: dynamic contrast-enhanced; FDG: ^18^F-fluoro-2-deoxy-d-glucose; LCC: large cell carcinoma; MIP: maximum intensity projection; MTT: mean transit time; MVD: microvessel density; NET: neuroendocrine tumor; NSCLC: non-small cell lung carcinoma; PACS: picture archiving and communication system; PET: positron emission tomography; PETvol: metabolic tumor volume; r: correlation coefficient; r2: correlation coefficient of determination; ROI: region of interest; SCLC: small cell lung carcinoma; SCC: squamous cell carcinoma; SD: standard deviation; SPV: standardized perfusion value; SUV: standardized uptake value; SUVmax: maximum standardized uptake value; SUVmean: mean standardized uptake value; TLG: total lesion glycolysis; VOI: volume of interest.

## Competing interests

PVH received IIS grants from Bayer Healthcare and Siemens Healthcare for CTP studies. However, the IIS grants from Siemens Healthcare do apply for other studies than the one presented here. All other authors declare that they have no competing interests.

## Authors’ contributions

The authors contributed to the work as follows: the study concepts; study design; data acquisition, analysis, and interpretation; manuscript drafting, and manuscript final version approval were done by all authors. Literature research was done by MWH, TDC, and PVH. The clinical studies were conducted by MWH, TDC, PG, CP, and PVH. The statistical analysis was performed by BS. Manuscript editing was done by MWH, TDC, RW, CP, JD, BS, KS, and PVH.

## Authors’ information

MWH, KS, and PVH are radiologists and nuclear medicine physicians with clinical and scientific interest in integrated multimodal imaging in oncology.
